# Long-term and trans-generational effects of neonatal experience on sheep behaviour

**DOI:** 10.1098/rsbl.2014.0273

**Published:** 2014-07

**Authors:** Corinna Clark, Joanna Murrell, Mia Fernyhough, Treasa O'Rourke, Michael Mendl

**Affiliations:** School of Veterinary Science, University of Bristol, Langford House, Langford BS40 5DU, UK

**Keywords:** sheep, early life experience, pain, animal welfare

## Abstract

Early life experiences can have profound long-term, and sometimes trans-generational, effects on individual phenotypes. However, there is a relative paucity of knowledge about effects on pain sensitivity, even though these may impact on an individual's health and welfare, particularly in farm animals exposed to painful husbandry procedures. Here, we tested in sheep whether neonatal painful and non-painful challenges can alter pain sensitivity in adult life, and also in the next generation. Ewes exposed to tail-docking or a simulated mild infection (lipopolysaccharide (LPS)) on days 3–4 of life showed higher levels of pain-related behaviour when giving birth as adults compared with control animals. LPS-treated ewes also gave birth to lambs who showed decreased pain sensitivity in standardized tests during days 2–3 of life. Our results demonstrate long-term and trans-generational effects of neonatal experience on pain responses in a commercially important species and suggest that variations in early life management can have important implications for animal health and welfare.

## 1. Introduction

Challenging experiences during early life can have profound long-term effects on an individual's phenotype and, in some cases, on that of its offspring. Effects of early stress on later metabolic function and stress reactivity, including across generations, have been studied in detail [1,2]. Early life influences on later pain sensitivity also warrant attention because variation in sensitivity may affect an individual's ability to cope with injury or disease and hence its vulnerability to suffering and poor welfare. There is evidence that painful neonatal experiences can influence the development of nociceptive systems and associated behaviour although, depending on the specifics of the early experience and the readout measures used [3], the direction of effects may vary (e.g. increased pain sensitivity in injured neonates (humans [4] and rats [5]); increased [6] and decreased [7] pain sensitivity in rats exposed to neonatal inflammatory pain). Perinatal experience of both central and peripheral lipopolysaccharide (LPS)-induced inflammation in the absence of overt pain, as may occur during infectious disease, also results in later changes in pain responsiveness, often hyperalgesia [8,9]. Thus, both painful and non-painful early life challenges can alter subsequent nociceptive processing. However, whether such effects carry over across generations is, to our knowledge, currently unknown.

Two recent studies of farm animals, for whom early pain and stress (e.g. tail-docking, neonatal infection) are common real-life events, have demonstrated short to medium-term effects on pain responsiveness. Pre-natal stress, induced by social mixing of pregnant sows during the second trimester, resulted in more pronounced nociceptive responses of piglets to tail-docking on day 3 of life [10], but increased nociceptive thresholds following capsaicin injection at *ca* eight weeks of life [11]. However, effects lasting into adult life and across generations have not been reported.

Here, we investigate in sheep whether painful and non-painful challenges often experienced by lambs (tail-docking without analgesia; infection (simulated by LPS challenge)), alter pain sensitivity in adult life and also in the next generation. Both challenges induce a physiological stress response in neonatal lambs [12]. We focus our investigation on the lambs' responses as adults to their first parturition, because parturition is a naturally occurring painful event and responses to it may influence ease of birth with potential consequences for offspring health and survival [13]. We also measure pain sensitivity in lambs of the next generation using standard nociceptive threshold testing [12].

## 2. Material and methods

The subjects were 20 Suffolk × Mule ewes and their first lamb(s). When the ewes were born, they were allocated to one of three early-life-challenge treatment groups balanced for birth weight and with siblings receiving different treatments. One group was tail-docked when 72–96 h old using a standard husbandry method: rubber ring elastration without analgesia (TD; *n* = 7). One group received an acute immune challenge when 48–72 h old using low dose LPS (*Escherichia coli* serotype 0127 : B8, Sigma-Aldrich, 0.2 μg kg^−1^, diluted 1 μg ml^−1^ in saline, i.v.), inducing a 1°C increase in temperature for up to 4 h (LPS; *n* = 6) [12]. Control ewes received no treatment (CONT; *n* = 7), but were handled for the same length of time as the treatment groups and underwent the same testing procedures.

With the exception of brief periods of housing at eight weeks and four months, all subjects were kept at grazing from 10 days old, put to a Hampshire ram when *ca* 18–20 months old and brought inside for lambing approximately one month before expected lambing date. They were housed in individual pens (1.8 × 1.8 m) one week prior to expected lambing date and gave birth to their first offspring at *ca* 24 months of age. Further details are provided in the electronic supplementary material.

Ewe behaviour during the 2 h prior to parturition was recorded on digital video and subsequently coded focusing on behaviours that may be indicative of stress and pain during parturition [14], and on the duration and ease of parturition [15]. Lists of behaviours recorded are provided in the electronic supplementary material.

Lambs were subjected to mechanical nociceptive threshold (MNT) tests on the morning and afternoon of day 2 of life, and on the morning of day 3. All lambs were tail-docked, and males were castrated, using rubber ring elastration on the afternoon of day 3. MNTs were measured 3 h later. At 2 h, 24 h and 3 days post-partum, lamb weights and rectal temperatures were recorded. MNTs were obtained using a pressure-driven analgesiometer as described previously [12]. Further details are provided in the electronic supplementary material.

All statistical analyses were carried out using IBM SPSS Statistics 19. Total frequencies of each behaviour occurring during the 2 h prior to parturition were analysed. Normally distributed data were analysed using one-way ANOVA for behaviour occurring prior to parturition (treatment (LPS, TD, CONT) as a fixed factor), and mixed-model repeated-measures GLM for MNT, weight and rectal temperature data (treatment (and lamb sex) as the between-subjects factor(s), and time of testing as the within-subjects factor). For mixed-model repeated-measures GLMs, the assumption of sphericity was tested using Mauchly's test and, if violated, Greenhouse–Geisser corrections were used. Non-normally distributed data were analysed using non-parametric statistics (e.g. Kruskal–Wallis tests; Mann–Whitney *U*-tests).

## 3. Results

The number of ewes assisted during lambing, the distribution of twins and singletons and litter sex ratios did not differ between treatments (see the electronic supplementary material).

**Figure 1.**
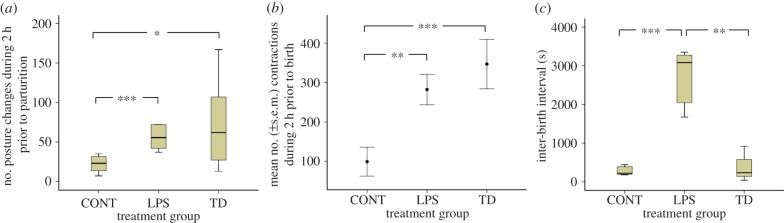
(*a*) Boxplot (median, inter-quartile range and range) of number of ewe posture changes during 2 h prior to parturition, (*b*) mean (±s.e.m.) number of contractions during 2 h prior to parturition and (*c*) boxplot of interval(s) between birth of first and second lamb, for ewes from the CONT, LPS and TD treatment groups. Post hoc test significant differences: *p ≤ 0.10; **p ≤ 0.05; ***p ≤ 0.01. (Online version in colour.)

The number of postural changes observed during the 2 h prior to parturition differed between treatments (Kruskal–Wallis *χ*^2^ = 6.458, *n* = 20, d.f. = 2, *p* = 0.04; figure 1*a*). CONT ewes made fewer postural changes than LPS (*U* = 39, *n* = 13, *p* = 0.01) and TD (*U* = 38, *n* = 14, *p* = 0.085) ewes, while TD and LPS ewes did not differ. The number of contractions observed also differed between treatments (*F*_2,17_ = 7.297, *p* = 0.005; figure 1*b*). CONT ewes exhibited significantly fewer contractions than both LPS ewes (Tukey HSD post hoc, *p* = 0.045) and TD ewes (Tukey HSD post hoc, *p* = 0.005), while TD and LPS ewes did not differ. There was a non-significant tendency for treatment to affect tail-wagging frequency (Kruskal–Wallis *χ*^2^ = 5.43, *n* = 20, d.f. = 2, *p* = 0.066), with tail-wagging being higher in TD ewes (median = 9; *U* = 41, *n* = 14, *p* = 0.034) than in CONT ewes (median = 3), and tending to be higher in LPS ewes (median = 7; *U* = 33.5, *n* = 13, *p* = 0.072) than CONT ewes. No treatment effects were detected for other behavioural categories (see the electronic supplementary material).

Inter-birth intervals between twin lambs were significantly affected by treatment (Kruskal–Wallis *χ*^2^ = 8.58, *n* = 13, d.f. = 2, *p* = 0.014; figure 1*c*). LPS ewes had significantly longer inter-birth intervals than both CONT ewes (*U* = 25, *n* = 10, *p* = 0.009) and TD ewes (*U* = 0, *n* = 8, *p* = 0.025), while TD and CONT ewes did not differ (*U* = 8, *n* = 8, *p* = 0.88). Lamb weights and temperatures were not affected by treatment (see the electronic supplementary material).

**Figure 2.**
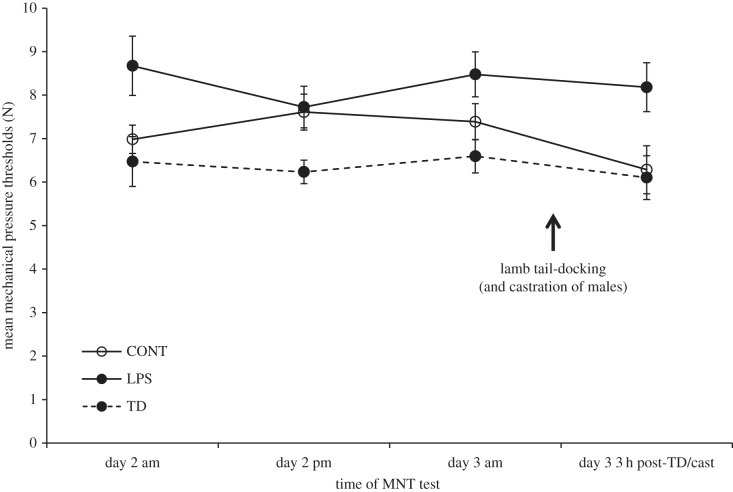
Mean (±s.e.m.) MNTs (N) across 2 days of testing in lambs from CONT, TD and LPS ewes. Day 3 pm testing took place 3 h after lamb tail-docking (and castration (cast) of males).

Lamb MNTs across day 2 and 3 post-partum were affected by treatment (*F*_2,27_ = 5.487, *p* = 0.01; figure 2). Lambs from LPS ewes showed significantly higher MNTs than lambs from TD ewes (Tukey HSD post hoc, *p* = 0.004) and a trend for higher MNTs than lambs from CONT ewes (Tukey HSD post hoc, *p* = 0.068), while CONT and TD lambs did not differ (Tukey HSD post hoc, *p* = 0.376). MNTs were not affected by lamb sex or by time of testing (day 2 am/pm, day 3 am/pm) indicating that tail-docking and castration did not markedly alter MNTs. There were no significant interaction effects (*p* > 0.2).

## 4. Discussion

Our results indicate that a female sheep's neonatal experience of painful and non-painful challenges can have long-term effects on its responses as an adult to a naturally occurring painful event, parturition, and on the pain sensitivity of its own offspring. Ewes experiencing TD or LPS showed more frequent postural changes and visible contractions and tended to show more tail-wagging in the 2 h prior to birth of their first lamb, than did control ewes. LPS ewes also showed a longer inter-birth interval between first and second lambs than control or TD ewes. An increased contraction rate and longer inter-birth intervals indicate enhanced straining which may reflect greater pain during parturition [15]. Tail-wagging is considered to be an indicator of pain in other species [16], as are postural changes [14]; increases in these behaviours are commonly observed in cows experiencing dystocia which is likely to be especially painful [15,17].

Overall, our results suggest that TD and LPS ewes experienced more painful births than CONT ewes. This may be because they were more sensitive to painful stimuli, in line with findings from humans and rats that simulated infection [8,9] and injurious tissue damage [4,5] during early life are linked with later hyperalgesia, perhaps via enhanced proinflammatory cytokine influences on prostaglandin function in pain processing pathways [8] and/or alterations in centrally mediated neuroimmune responses [5]. It is also possible that early experience affected factors that influence birth ease (e.g. disproportionate fetus size, uterine activity) with knock-on effects on pain responses, although lamb birth weights did not differ between treatments.

Our findings also indicate a trans-generational effect on pain sensitivity of a brief simulated infection during early life. Neonatal offspring of LPS-treated ewes showed hypoalgesic responses to repeated MNT relative to TD and CONT ewes. This effect may have been mediated by heritable epigenetic changes (e.g. DNA methylation) induced in ewes by early life experience and transmitted to offspring via the germline [1]. Alternatively, non-genomic mechanisms may have been responsible. Offspring of LPS ewes probably experienced more difficult births owing to increased contractions and prolonged inter-birth intervals. This birth stress may have induced hypoalgesia as observed in other species [18], including humans, where infants exposed to the intense pressures and associated pain of vaginal delivery show reduced postnatal pain sensitivity compared with those delivered by Caesarean section [19]. In rats, oxytocin may be one mediator of this effect, and stress-induced activation of other systems (e.g. opioids, catecholamines) may also play a role [18]. However, TD ewes also experienced increased contractions, although not increased inter-birth intervals, and their lambs did not show hypoalgesia, suggesting the influence of factors specifically related to LPS experience.

Our results demonstrate long-term effects of neonatal experience on responses to a real-life pain challenge in a commercially important species. They also indicate that such experience may have trans-generational effects on the pain sensitivity of offspring born 2 years later. Further research is required to confirm these findings and elucidate potential underlying mechanisms. In particular, the potential for trans-generational effects of early experience warrants attention. Such effects may vary between species and render them more or less adaptable in the face of differing early life events. Overall, our findings emphasize the impact that events during the perinatal period can have on an individual's life history and suggest that variations in management of early life health and husbandry conditions can have important long-term implications for animal health and welfare.

## Supplementary Material

Supplementary information on methods and results

## Notes

The research was conducted under UK Home Office licence 30/2420.

## Data accessibility

The data supporting this article are accessible in the Dryad Digital Repository (doi:10.5061/dryad.83rv1).
