# Supplementary material for: Long-term and trans-generational effects of neonatal experience on sheep behaviour
Source: Biol Lett. 2014 Jul;10(7):20140273. doi: 10.1098/rsbl.2014.0273 (PMC4126620; doi:10.1098/rsbl.2014.0273)
Supplement: Supplementary information on methods and results [file rsbl20140273supp1.docx]

**Long-term and trans-generational effects of neonatal experience on sheep behaviour**

**Electronic supplementary material (ESM)**

Corinna Clark, Joanna Murrell, Mia Fernyhough, Treasa O’ Rourke and Michael Mendl

School of Veterinary Science, University of Bristol, Langford House, Langford, BS40 5DU, UK

This document contains supporting information on Methods and Results.

**1. Supplementary information on methods**

**(a) Housing and care of ewes at parturition**

The subjects were 20 Suffolk x Mule ewes and their first lamb/s. Three of the ewes were singletons, four were from female twin pairs, 11 from male-female twin pairs, and two from a set of all female triplets. Siblings were assigned to different treatment groups. All subject animals were kept at the University of Bristol from birth up until, and throughout, their first pregnancy. Once housed in individual pens one week prior to their expected lambing date, all ewes received hay and water *ad libitum* with supplementary concentrate given twice daily (8am and 6pm). Lactating ewes were given additional supplementary alfalfa at 8am and 8pm daily. Ewes in individual pens were monitored 24 hours a day in order to detect the onset of lambing. When nest-building behaviour, discharge at the vulva, appearance of amniotic sac, or straining behaviour was detected, digital cameras (Sony Handycam Carl Zeiss Vario-Tessar Optical Zoom 40X) were installed over the pen to record behaviour until 2 hours post-partum. Lambing assistance was given if amniotic fluids had been seen but there was no progress (no lamb parts visible) after 1 hour, or if after 30 minutes of active straining no progress had been made.

**(b) Behaviour recording**

Video-recordings of behaviour during the 2h prior to parturition were analysed. Behaviour was coded using Observer v.XT 7.0 (Noldus). The focus of data collection was on behaviours that may be indicative of stress and pain during parturition in the ewe, and on the duration and ease of parturition. Frequencies of the following behaviours were recorded: postural shifts (shifts between: normal standing, standing with legs stretched, standing in a squatting position, resting on knees, dog-sitting, ventral lying with legs tucked in, ventral lying with partial extension of hind legs, lateral lying); ear posture changes (one or both ears move); visible contractions; open-mouthed bleat vocalizations; closed-mouth vocalizations; turning to face hind quarters; touching the nose to the flank; tail-wagging. Durations spent in the main postural categories (standing, ventral lying, lateral lying, other (dog-sitting or resting on knees)) were recorded and converted into proportions of observation time.

**(c) Mechanical nociceptive threshold (MNT) testing**

MNTs were obtained using a pressure-driven analgesiometer (Topcat Metrology Ltd., Ely, Cambs, UK) consisting of a lightweight pneumatic actuator that pushed three rounded metal pins (each tipped with a 2.5mm diameter ball bearing) against the subject’s limb. The cuff was fixed securely around the hindlimb of the subject below the hock joint, so that the three pins were in contact with the lateral aspect of the metatarsal bone. The actuator was inflated manually from a syringe, with a ‘traffic light’ system (green signalling to press harder; red signalling to decrease pressure) ensuring that the operator stayed within the inflation rate window (approximately 0.87 N/sec). The behavioural end-points that signalled threshold responses were: lifting the leg in response to the stimulus; shifting of weight on the hind legs in response to the stimulus (without lifting feet off the ground); moving away from and in response to the stimulus. Once a behavioural end-point had been detected, a handset button was pressed to record the threshold force in Newtons, and the pressure was released instantly. Each subject’s left hind leg was tested first, followed by the right hind leg. The purpose of testing both legs was to ensure that the threshold value was consistent and accurate. Responses within 1 N were deemed to be consistent, and the mean score across both legs was used as the threshold value. Where consistency was not found between consecutive left and right leg tests, the test was repeated at approximately 2 minute intervals up to a maximum of 3 times per leg. Subjects were unrestrained during testing, but prevented from feeding.

**2. Supplementary information on results**

**(a) Assistance during parturition, and litter composition**

Seven ewes were given assistance during lambing, but this did not differ across treatment groups (Fisher’s exact: 2.331, p=0.381). A total of 33 lambs (19 male, 14 female) were born with no deaths. There were 13 sets of twins and 7 singletons, with no difference in the distribution of these across treatments (Fisher’s exact: 2.331, p=0.381). Likewise, the sex ratio of each litter (male/(male + female)) did not differ between treatments (Fisher’s exact: 1.715, p=0.940).

**(b) Ewe behaviour prior to parturition**

No significant effect of treatment was found for several behaviour patterns. The overall proportion of time spent in each of the main postural categories did not differ between the treatment groups (standing: F_2,17_=1.966, p=0.171; ventral lying: F_2,17_=1.292, p=0.30; lateral lying: F_2,17_=0.674, p=0.523; other (dog-sitting and resting on knees): Kruskal-Wallis chi-square=0.084, n=20, df=2, p=0.959). The number of ewe ear posture changes during the 2h preceding parturition did not differ between treatments (F_2,17_=0.760, p=0.483), and nor did the number of open mouth bleats (Kruskal-Wallis chi-square=0.027, n=13, df=2, p=0.987) or closed-mouth vocalizations (Kruskal-Wallis chi-square=0.068, n=13, df=2, p=0.967), although sample sizes were reduced due to problems with sound recording from some videos. There were no effects of treatment on the frequency with which ewes turned to face their hind quarters (F_2,17_=1.114, p=0.351), or touched nose to flank (Kruskal-Wallis chi-square=1.38, n=20, df=2, p=0.5).

**(c) Lamb weight and rectal temperature**

Lamb weights and temperatures were not affected by ewe treatment or lamb gender, and there were no interaction effects (p>0.3). Lambs grew heavier between 2h and 3days post-partum (F_1.4,39.1_=301.08, p<0.001) and their rectal temperatures decreased during this time (F_1.5,40.4_=13.38, p<0.001).
